# Steps towards equity in research

**DOI:** 10.1017/cts.2023.8

**Published:** 2023-03-03

**Authors:** Robert D. Sege, Danielle Laraque-Arena

**Affiliations:** 1 Institute for Clinical Research and Health Policy Studies, Tufts Medical Center, Boston, MA, USA; 2 New York Academy of Medicine, New York, NY, USA; 3 Mailman School of Public Health and Vagelos College of Physicians & Surgeons, Columbia University, New York, NY, USA

## Steps towards Equity in Research

Health disparities in the United States of America (USA) contribute to relatively low and declining life expectancy as compared to other OECD countries [1–4]. We explore how clinical translational research can address health equity, beginning with an understanding of how research has failed to address racial disparities in health and concluding with feasible actions to redress research practices.

## What Research Is Done?

Research concerning diseases that primarily affect marginalized US populations are underfunded and understudied. Farooq reported that research funding related to sickle cell disease (SSD), the most common monogenic disease in the USA, amounts to under $1000/affected person, while cystic fibrosis (CF) funding (the most common monogenic disease among White Americans) is over $10,000/person. Research investment matters: individuals with CF have new treatments and near-normal life expectancy, while advances in treatment for SSD and life expectancy have lagged [5].

Examples of inequitable funding abound: research on triple-negative breast cancer, common among African American women, is relatively underfunded. At least 200,000 US adults, primarily Black and Latina women, have Systemic Lupus Erythematosus; however, in 2021 NIH set aside less than $900/affected person. Firearms injuries, the leading cause of death in children and adolescents [6,7], disproportionately affect young Black men, but until recently federal law effectively prohibited public health studies to reduce firearms injuries.

## Who Does the Research and How Is It Funded?

Underrepresentation of Black scientists may underlie the problems in studying issues relevant to Black Americans. Black investigators account for only about 2% of NIH-funded Principal Investigators.

Felicia Marie Knaul, economist and researcher of global health and breast cancer, points to research funding fueled by distinct personal interest and siloed fundraising resulting in missed opportunities to address systemic issues [8].

Hoppe noted 3 problem areas that lead to disparate peer-review outcomes: decision to discuss, impact score assignment, and topic choice [9]. Black applicants chose topics such as community and population health that receive lower award rates than investigator-initiated proposals at earlier translational stages. Study sections, drawn from the ranks of funded investigator, may have less enthusiasm for research focused on minoritized communities or relevance to public health or health disparities.

## Research Participation

Clinical research involves the participation of affected individuals. Black Americans are far less likely to enroll in research trials than would be expected based on disease prevalence [10]. Although better framing of randomized controlled trials and medical professional training may help [11,12], the distrust of research may be well justified. Recent papers have suggested that Black and White research participants are treated differently within the same trials [13].

## Results Reporting

Knowledge of health disparities depends on data availability. A recent analysis demonstrated that only 38% of published articles describing clinical trials for cancer drug approvals indicated the proportion of research participants who were Black [10]. Research reports should expand the use of the ten-year-old PRISMA Equity guidelines [14] or the recent International Journal editors’ recommendations [15].

## A Pathway to Equity through Translational Research Redesign

Clinical translational science can diversify research teams, match research priorities with health needs, improve diverse research participation, and follow guidelines for results reporting.

This perspective echoes a recent NAM Perspective plan of action for dismantling racism in the research endeavor [16]. The recommendations are anchored on the principle of addressing leading causes of death throughout the life course and the training of medical professionals in socially accountable, competency-based educational models [6,7,17,18]. The lack of diversity in the entire research workforce can be addressed through programs that improve access to careers as independent investigators and engage community residents as co-researchers [19].

## Addressing the Urgent Need for Change

### What NIH Can Do?

Effective efforts towards diversity of the educational pipeline will have delayed effects because the median age of new NIH R01 investigators is 44 years old. However, as suggested by Selker, the clinical translational science can make concerted efforts now to include individuals from marginalized communities in research as part of broadly engaged research teams [20]. This broadly engaged team science model offers a paradigm shift that can be instituted in clinical translational science.

We also propose a multi-pronged approach that includes needed competencies that support understanding the burden of disease for diverse US and global populations, adopting community accountability approaches that advance equity in research [17], and hiring diverse scholars and research staff.

Within the clinical translational science training programs, we can ensure that our trainees graduate equipped with tools to identify, understand, and address issues related to health disparities. These tools extend standard training in biostatistics, epidemiology, and ethics with skills related to social epidemiology, use of qualitative and quantitative measures, exposure to metrics of inequality, critical tools for evaluating data sources, and experience in reporting research results in standard recommended formats that promote equity [14,15]. In our personal experience, trainees are very interested in this emerging field of study.

Lastly, building trust between the research community and the public requires a change in mindset among investigators to include issues of health equity in their work, improvements in science communication, and the creation of meaningful community advisory boards for clinical translational science institutes. The enormous investment in biomedical and translational research has accelerated the health impact of the recent explosion in scientific knowledge. Attention to health disparities can help translate these advances to improvements in health and longevity.
